# 4-Chloro-2-((1*R*)-1-{[(*R*)-(2-chlorophen­yl)(cyclo­pent­yl)meth­yl]amino}prop­yl)phenol

**DOI:** 10.1107/S1600536808042025

**Published:** 2008-12-13

**Authors:** Guang-You Zhang, Ting Yang, Bao-Wang Xu, Di-Juan Chen, Wan-Hui Wang

**Affiliations:** a School of Chemistry, Jinan University, 250022, People’s Republic of China; b Qilu Pharmaceutical Co Ltd, Shandong Provience, 250100, People’s Republic of China; c Graduate School of Science and Engineering, Saitama University, Sakura, Saitama 338-8570, Japan

## Abstract

In the title compound, C_21_H_25_Cl_2_NO, the dihedral angle between the two benzene rings is 33.18 (11)°. The five-membered ring adopts an envelope conformation. There is an intra­molecular O—H⋯N hydrogen bond. In the crystal, mol­ecules are linked by weak N—H⋯Cl hydrogen bonds, forming a helical chain along the *c* axis.

## Related literature

For related literature on amino­phenols, see: Cimarelli *et al.* (2002 ▶); Joshi & Malhotra (2003 ▶); Li *et al.* (2004 ▶); Puigjaner *et al.* (1999 ▶); Watts *et al.* (2005 ▶). For the synthesis, see: Yang *et al.* (2005 ▶).
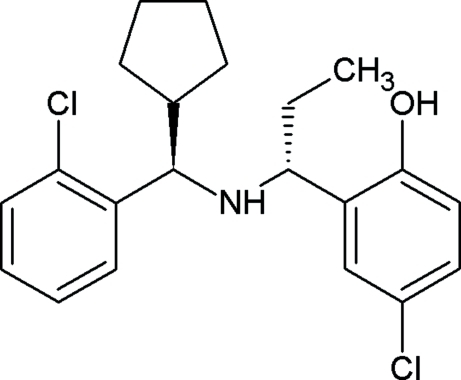


## Experimental

### 

#### Crystal data

C_21_H_25_Cl_2_NO
*M* *_r_* = 378.32
Orthorhombic, 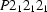

*a* = 10.9802 (17) Å
*b* = 11.5607 (18) Å
*c* = 15.536 (2) Å
*V* = 1972.1 (5) Å^3^
*Z* = 4
Mo *K*α radiation
μ = 0.34 mm^−1^
*T* = 298 (2) K
0.49 × 0.45 × 0.38 mm

#### Data collection

Bruker SMART APEX CCD area-detector diffractometer
Absorption correction: multi-scan (**SADABS**; Sheldrick, 1996 ▶) *T*_min_ = 0.852, *T*_max_ = 0.882
10332 measured reflections
3647 independent reflections
3266 reflections with *I* > 2σ(*I*)
*R*_int_ = 0.025

#### Refinement

*R*[*F* ^2^ > 2σ(*F* ^2^)] = 0.035
*wR*(*F* ^2^) = 0.093
*S* = 1.03
3647 reflections
231 parameters
1 restraint
H atoms treated by a mixture of independent and constrained refinement
Δρ_max_ = 0.17 e Å^−3^
Δρ_min_ = −0.20 e Å^−3^
Absolute structure: Flack (1983 ▶), 1556 Friedel pairs
Flack parameter: 0.06 (6)

### 

Data collection: *SMART* (Bruker, 1999 ▶); cell refinement: *SAINT* (Bruker, 1999 ▶); data reduction: *SAINT*; program(s) used to solve structure: *SHELXS97* (Sheldrick, 2008 ▶); program(s) used to refine structure: *SHELXL97* (Sheldrick, 2008 ▶); molecular graphics: *SHELXTL* (Sheldrick, 2008 ▶); software used to prepare material for publication: *SHELXTL*.

## Supplementary Material

Crystal structure: contains datablocks I, global. DOI: 10.1107/S1600536808042025/is2364sup1.cif

Structure factors: contains datablocks I. DOI: 10.1107/S1600536808042025/is2364Isup2.hkl

Additional supplementary materials:  crystallographic information; 3D view; checkCIF report

## Figures and Tables

**Table 1:** Hydrogen-bond geometry (Å, °)

*D*—H⋯*A*	*D*—H	H⋯*A*	*D*⋯*A*	*D*—H⋯*A*
N1—H1⋯Cl2^i^	0.848 (19)	2.913 (13)	3.7023 (18)	156 (2)
O1—H1*A*⋯N1	0.82	1.93	2.642 (2)	144

Symmetry code: (i) 
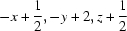
.

**Fig. 1.**
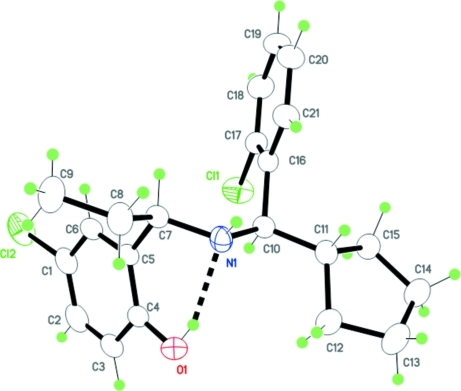
The asymmetric unit of (I), showing the atom-labelling schemes. Displacement ellipsoids are drawn at the 30% probability level and H atoms are shown as spheres of arbitrary radii.

**Fig. 2.**
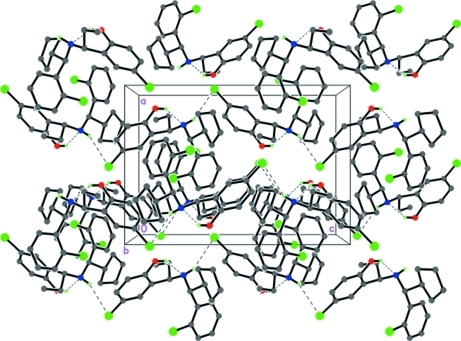
A packing diagram of (I), view down the b axis, showing the formation of helical chains through O1—H1A···N1 and N1—H1···Cl2 hydrogen bonds (dashed lines). H atoms not involved in hydrogen bonds have been omitted.

## supplementary crystallographic information

### Comment

The synthesis of enantiopure aminophenols that have different functionalities is an important subject of research because compounds of this class are widespread in natural products, show pharmacological activity and have recently found application in asymmetric synthesis as chiral bases, auxiliaries and ligands (Cimarelli *et al.*, 2002). Chiral aminophenols which are similar to amino alcohols have attracted wide attention for the reason that they can be used in catalytic asymmetric reactions (Puigjaner *et al.*, 1999; Li *et al.*, 2004; Watts *et al.*, 2005), which is one of the most active areas of research in organic chemistry (Joshi & Malhotra, 2003). The synthesis of new aminoalkylphenols is therefore of interest because of potential as asymmetric catalysts.

As part of our continuing studies of chiral aminophenols, we now report the crystal structure of the title compound, (I), which was intially prepared to test its asymmetric catalytic activity. These compounds were prepared by conventional condensation of (*R*)-1-(2-chlorophenyl)-1-cyclopentylmethanamine with 1-(5-chloro-2-hydroxyphenyl)ethanone, followed by reduction using sodium borohydride in a tetrahydrofuran-ethanol (1:1 *v*/*v*) mixture. An X-ray study of the title compound, (I), was carried out and the results are presented here. The molecular structure of (I) is shown in Fig. 1.

The molecule has two chiral centres (C7/C10), which have configuration *R*, *R*, as shown in Fig. 1. In the molecules of (I), the five-membered rings adopts an envelope conformation. The dihedral angle between the benzene rings is 33.18 (11)°. There is an intramolecular O1—H1A···N1 hydrogen bond (Table 1). Phenol atom O1 acts as a hydrogen bond donor to atom N1, with O1···N1 = 2.647 (2) Å, which indicates a comparatively strong intramolecular hydrogen bond (Table 1); this distance is significantly shorter than the sum (3.07 Å) of the van der Waals radii for N and O atoms. The molecules are linked *via* N1—H1···Cl2 hydrogen bonds. An interesting feature of the structure is that the N1—H1···Cl2 hydrogen-bond gives rise to a spiral chain of molecules along the *c* direction. There are no π-π stacking interactions are present in the structure of (I).

### Experimental

The title compound were prepared according to the procedure of Yang *et al.* (2005). (*R*)-1-(2-chlorophenyl)-1-cyclopentylmethanamine (0.9 mmol) and 1-(5-chloro-2-hydroxyphenyl)propan-1-one (0.9 mmol) were dissolved in methanol (10 ml) and reacted at room temperature for 48 h. After removal of the solvent, NaBH_4_ (4.5 mmol) was added to the solution in THF/ethanol (1:1 *v*/*v*, 20 ml) and stirred at 273 K until the solution became colourless. The solvent was then removed under reduced pressure. Water (10 ml) was added to the residue and 1 N HCl was added dropwise until hydrogen production ceased. The mixture was neutralized with aqueous Na_2_CO_3_, then extracted with CHCl_3_, and the organic layer was dried over anhydrous sodium sulfate. The solvent was removed under reduced pressure. Further purification was carried out by thin-layer silica-gel chromatography (chloroform) to give a colourless solid (yield 80.5%). Crystals of (I) were grown from a n-hexane solution.

### Refinement

The N-bound H atom was located in a Fourier difference map and was refined with a distance restraint of N—H = 0.86 (1) Å, and with *U*_iso_(H) = 1.2*U*_eq_(N). The O-bound and C-bound H atoms were positioned geometrically (O—H = 0.82 Å and C—H = 0.93–0.98 Å) and were treated as riding, with *U*_iso_(H) = 1.2*U*_eq_ (C) or 1.5*U*_eq_(O, methyl C).

### Crystal data

**Table:**

C_21_H_25_Cl_2_NO	*F*(000) = 800
*M**_r_* = 378.32	*D*_x_ = 1.274 Mg m^−^^3^
Orthorhombic, *P*2_1_2_1_2_1_	Mo *K*α radiation, λ = 0.71073 Å
Hall symbol: P 2ac 2ab	Cell parameters from 4568 reflections
*a* = 10.9802 (17) Å	θ = 2.2–25.5°
*b* = 11.5607 (18) Å	µ = 0.34 mm^−^^1^
*c* = 15.536 (2) Å	*T* = 298 K
*V* = 1972.1 (5) Å^3^	Block, colourless
*Z* = 4	0.49 × 0.45 × 0.38 mm

### Data collection

**Table:**

Bruker SMART APEX CCD area-detector diffractometer	3647 independent reflections
Radiation source: fine-focus sealed tube	3266 reflections with *I* > 2σ(*I*)
graphite	*R*_int_ = 0.025
φ and ω scans	θ_max_ = 25.5°, θ_min_ = 2.2°
Absorption correction: multi-scan (*SADABS*; Sheldrick, 1996)	*h* = −12→13
*T*_min_ = 0.852, *T*_max_ = 0.882	*k* = −13→13
10332 measured reflections	*l* = −18→15

### Refinement

**Table:**

Refinement on *F*^2^	Secondary atom site location: difference Fourier map
Least-squares matrix: full	Hydrogen site location: inferred from neighbouring sites
*R*[*F*^2^ > 2σ(*F*^2^)] = 0.035	H atoms treated by a mixture of independent and constrained refinement
*wR*(*F*^2^) = 0.093	*w* = 1/[σ^2^(*F*_o_^2^) + (0.0504*P*)^2^ + 0.2043*P*] where *P* = (*F*_o_^2^ + 2*F*_c_^2^)/3
*S* = 1.03	(Δ/σ)_max_ = 0.001
3647 reflections	Δρ_max_ = 0.17 e Å^−^^3^
231 parameters	Δρ_min_ = −0.20 e Å^−^^3^
1 restraint	Absolute structure: Flack (1983), 1556 Friedel pairs
Primary atom site location: structure-invariant direct methods	Flack parameter: 0.06 (6)

### Special details

**Table:**

Geometry. All e.s.d.'s (except the e.s.d. in the dihedral angle between two l.s. planes) are estimated using the full covariance matrix. The cell e.s.d.'s are taken into account individually in the estimation of e.s.d.'s in distances, angles and torsion angles; correlations between e.s.d.'s in cell parameters are only used when they are defined by crystal symmetry. An approximate (isotropic) treatment of cell e.s.d.'s is used for estimating e.s.d.'s involving l.s. planes.
Refinement. Refinement of *F*^2^ against ALL reflections. The weighted *R*-factor *wR* and goodness of fit *S* are based on *F*^2^, conventional *R*-factors *R* are based on *F*, with *F* set to zero for negative *F*^2^. The threshold expression of *F*^2^ > σ(*F*^2^) is used only for calculating *R*-factors(gt) *etc*. and is not relevant to the choice of reflections for refinement. *R*-factors based on *F*^2^ are statistically about twice as large as those based on *F*, and *R*- factors based on ALL data will be even larger.

### Fractional atomic coordinates and isotropic or equivalent isotropic displacement parameters (Å^2^)

**Table:**

	*x*	*y*	*z*	*U*_iso_*/*U*_eq_	
C1	0.4025 (2)	0.9204 (2)	0.46157 (14)	0.0567 (6)	
C2	0.3358 (3)	0.8241 (2)	0.43935 (15)	0.0695 (7)	
H2	0.3513	0.7853	0.3881	0.083*	
C3	0.2468 (3)	0.7864 (2)	0.49342 (16)	0.0687 (7)	
H3	0.2013	0.7216	0.4786	0.082*	
C4	0.2227 (2)	0.84331 (19)	0.57058 (15)	0.0576 (6)	
C5	0.29329 (17)	0.93830 (17)	0.59471 (12)	0.0442 (5)	
C6	0.38307 (18)	0.97536 (19)	0.53893 (13)	0.0476 (5)	
H6	0.4312	1.0384	0.5539	0.057*	
C7	0.26917 (18)	1.00349 (17)	0.67807 (13)	0.0453 (5)	
H7	0.3437	1.0439	0.6955	0.054*	
C8	0.1659 (2)	1.0917 (2)	0.66944 (15)	0.0621 (6)	
H8A	0.0917	1.0509	0.6542	0.075*	
H8B	0.1528	1.1279	0.7250	0.075*	
C9	0.1884 (3)	1.1847 (2)	0.60364 (17)	0.0757 (7)	
H9A	0.2633	1.2238	0.6167	0.113*	
H9B	0.1225	1.2393	0.6048	0.113*	
H9C	0.1937	1.1506	0.5475	0.113*	
C10	0.33131 (17)	0.84631 (17)	0.77983 (12)	0.0420 (4)	
H10	0.3589	0.7969	0.7324	0.050*	
C11	0.27940 (18)	0.76780 (18)	0.84923 (13)	0.0479 (5)	
H11	0.2531	0.8162	0.8976	0.057*	
C12	0.1718 (2)	0.6922 (2)	0.82169 (16)	0.0626 (6)	
H12A	0.0968	0.7366	0.8208	0.075*	
H12B	0.1855	0.6594	0.7650	0.075*	
C13	0.1665 (3)	0.5979 (3)	0.8899 (2)	0.0886 (9)	
H13A	0.1118	0.6201	0.9360	0.106*	
H13B	0.1376	0.5261	0.8649	0.106*	
C14	0.2942 (2)	0.5833 (2)	0.92374 (19)	0.0737 (7)	
H14A	0.3261	0.5080	0.9081	0.088*	
H14B	0.2951	0.5901	0.9860	0.088*	
C15	0.3705 (2)	0.67875 (19)	0.88315 (15)	0.0563 (5)	
H15A	0.4197	0.6482	0.8365	0.068*	
H15B	0.4240	0.7135	0.9256	0.068*	
C16	0.44189 (17)	0.91271 (16)	0.81295 (12)	0.0405 (4)	
C17	0.55907 (18)	0.89656 (17)	0.78285 (13)	0.0466 (5)	
C18	0.6565 (2)	0.9581 (2)	0.81494 (16)	0.0596 (6)	
H18	0.7342	0.9451	0.7932	0.071*	
C19	0.6393 (2)	1.0384 (2)	0.87866 (16)	0.0639 (6)	
H19	0.7048	1.0806	0.8999	0.077*	
C20	0.5237 (2)	1.0559 (2)	0.91091 (15)	0.0608 (6)	
H20	0.5112	1.1094	0.9547	0.073*	
C21	0.4275 (2)	0.99458 (18)	0.87846 (14)	0.0522 (5)	
H21	0.3501	1.0078	0.9007	0.063*	
Cl1	0.58898 (6)	0.79389 (6)	0.70334 (4)	0.0724 (2)	
Cl2	0.51106 (6)	0.97523 (8)	0.39044 (4)	0.0820 (2)	
N1	0.23430 (14)	0.92140 (16)	0.74620 (11)	0.0469 (4)	
H1	0.1982 (19)	0.9534 (18)	0.7882 (11)	0.056*	
O1	0.13051 (17)	0.80387 (17)	0.62012 (11)	0.0773 (5)	
H1A	0.1323	0.8364	0.6670	0.116*	

### Atomic displacement parameters (Å^2^)

**Table:**

	*U*^11^	*U*^22^	*U*^33^	*U*^12^	*U*^13^	*U*^23^
C1	0.0571 (12)	0.0642 (14)	0.0489 (12)	0.0175 (12)	−0.0106 (10)	0.0005 (10)
C2	0.100 (2)	0.0584 (14)	0.0502 (13)	0.0194 (14)	−0.0232 (14)	−0.0075 (11)
C3	0.102 (2)	0.0470 (12)	0.0576 (14)	−0.0064 (13)	−0.0373 (14)	0.0001 (12)
C4	0.0675 (14)	0.0481 (11)	0.0570 (13)	−0.0112 (11)	−0.0277 (11)	0.0128 (10)
C5	0.0439 (10)	0.0445 (11)	0.0442 (10)	0.0005 (9)	−0.0147 (8)	0.0040 (8)
C6	0.0458 (11)	0.0477 (11)	0.0491 (11)	0.0033 (9)	−0.0125 (9)	−0.0017 (9)
C7	0.0441 (11)	0.0458 (11)	0.0459 (10)	−0.0009 (8)	−0.0084 (8)	0.0015 (9)
C8	0.0641 (14)	0.0609 (13)	0.0614 (13)	0.0163 (12)	0.0008 (11)	0.0083 (11)
C9	0.100 (2)	0.0566 (14)	0.0705 (15)	0.0221 (15)	0.0023 (15)	0.0104 (12)
C10	0.0412 (10)	0.0434 (10)	0.0414 (10)	0.0026 (8)	−0.0012 (8)	−0.0017 (8)
C11	0.0450 (11)	0.0529 (12)	0.0457 (11)	0.0025 (10)	−0.0024 (8)	0.0038 (9)
C12	0.0482 (12)	0.0691 (14)	0.0706 (15)	−0.0085 (11)	−0.0056 (10)	0.0153 (13)
C13	0.0688 (16)	0.090 (2)	0.107 (2)	−0.0184 (15)	−0.0105 (16)	0.0458 (18)
C14	0.0747 (16)	0.0628 (14)	0.0837 (17)	−0.0079 (14)	−0.0110 (13)	0.0242 (14)
C15	0.0546 (12)	0.0533 (12)	0.0610 (13)	0.0016 (10)	−0.0084 (10)	0.0108 (10)
C16	0.0437 (10)	0.0379 (9)	0.0401 (10)	0.0049 (8)	−0.0029 (8)	0.0018 (8)
C17	0.0470 (11)	0.0439 (10)	0.0488 (11)	0.0027 (9)	−0.0002 (9)	0.0001 (9)
C18	0.0447 (11)	0.0578 (13)	0.0763 (15)	0.0009 (10)	−0.0047 (11)	0.0011 (12)
C19	0.0616 (14)	0.0532 (13)	0.0768 (16)	−0.0082 (11)	−0.0235 (12)	−0.0001 (12)
C20	0.0738 (16)	0.0472 (12)	0.0615 (13)	0.0045 (12)	−0.0138 (12)	−0.0097 (11)
C21	0.0520 (12)	0.0485 (12)	0.0562 (12)	0.0073 (10)	−0.0037 (10)	−0.0068 (9)
Cl1	0.0588 (3)	0.0828 (4)	0.0757 (4)	0.0041 (3)	0.0154 (3)	−0.0274 (3)
Cl2	0.0672 (4)	0.1215 (6)	0.0573 (3)	0.0189 (4)	0.0075 (3)	−0.0007 (4)
N1	0.0392 (9)	0.0552 (10)	0.0463 (9)	0.0065 (8)	−0.0023 (7)	0.0070 (8)
O1	0.0815 (12)	0.0804 (12)	0.0701 (11)	−0.0394 (10)	−0.0257 (10)	0.0165 (10)

### Geometric parameters (Å, °)

**Table:**

C1—C6	1.376 (3)	C11—C12	1.531 (3)
C1—C2	1.377 (4)	C11—H11	0.9800
C1—Cl2	1.745 (3)	C12—C13	1.521 (3)
C2—C3	1.360 (4)	C12—H12A	0.9700
C2—H2	0.9300	C12—H12B	0.9700
C3—C4	1.393 (4)	C13—C14	1.507 (4)
C3—H3	0.9300	C13—H13A	0.9700
C4—O1	1.351 (3)	C13—H13B	0.9700
C4—C5	1.395 (3)	C14—C15	1.522 (3)
C5—C6	1.381 (3)	C14—H14A	0.9700
C5—C7	1.522 (3)	C14—H14B	0.9700
C6—H6	0.9300	C15—H15A	0.9700
C7—N1	1.472 (3)	C15—H15B	0.9700
C7—C8	1.531 (3)	C16—C17	1.382 (3)
C7—H7	0.9800	C16—C21	1.399 (3)
C8—C9	1.504 (3)	C17—C18	1.378 (3)
C8—H8A	0.9700	C17—Cl1	1.744 (2)
C8—H8B	0.9700	C18—C19	1.370 (3)
C9—H9A	0.9600	C18—H18	0.9300
C9—H9B	0.9600	C19—C20	1.379 (3)
C9—H9C	0.9600	C19—H19	0.9300
C10—N1	1.470 (2)	C20—C21	1.369 (3)
C10—C11	1.520 (3)	C20—H20	0.9300
C10—C16	1.526 (3)	C21—H21	0.9300
C10—H10	0.9800	N1—H1	0.848 (19)
C11—C15	1.529 (3)	O1—H1A	0.8200
			
C6—C1—C2	120.7 (2)	C12—C11—H11	108.2
C6—C1—Cl2	119.43 (19)	C13—C12—C11	104.11 (19)
C2—C1—Cl2	119.89 (19)	C13—C12—H12A	110.9
C3—C2—C1	119.1 (2)	C11—C12—H12A	110.9
C3—C2—H2	120.5	C13—C12—H12B	110.9
C1—C2—H2	120.5	C11—C12—H12B	110.9
C2—C3—C4	121.1 (2)	H12A—C12—H12B	109.0
C2—C3—H3	119.4	C14—C13—C12	106.7 (2)
C4—C3—H3	119.4	C14—C13—H13A	110.4
O1—C4—C3	118.2 (2)	C12—C13—H13A	110.4
O1—C4—C5	121.9 (2)	C14—C13—H13B	110.4
C3—C4—C5	119.8 (2)	C12—C13—H13B	110.4
C6—C5—C4	118.2 (2)	H13A—C13—H13B	108.6
C6—C5—C7	120.31 (17)	C13—C14—C15	106.6 (2)
C4—C5—C7	121.45 (19)	C13—C14—H14A	110.4
C1—C6—C5	121.0 (2)	C15—C14—H14A	110.4
C1—C6—H6	119.5	C13—C14—H14B	110.4
C5—C6—H6	119.5	C15—C14—H14B	110.4
N1—C7—C5	109.75 (16)	H14A—C14—H14B	108.6
N1—C7—C8	107.43 (17)	C14—C15—C11	105.70 (18)
C5—C7—C8	112.60 (16)	C14—C15—H15A	110.6
N1—C7—H7	109.0	C11—C15—H15A	110.6
C5—C7—H7	109.0	C14—C15—H15B	110.6
C8—C7—H7	109.0	C11—C15—H15B	110.6
C9—C8—C7	114.5 (2)	H15A—C15—H15B	108.7
C9—C8—H8A	108.6	C17—C16—C21	116.28 (18)
C7—C8—H8A	108.6	C17—C16—C10	123.98 (17)
C9—C8—H8B	108.6	C21—C16—C10	119.74 (17)
C7—C8—H8B	108.6	C18—C17—C16	122.04 (19)
H8A—C8—H8B	107.6	C18—C17—Cl1	117.49 (16)
C8—C9—H9A	109.5	C16—C17—Cl1	120.46 (15)
C8—C9—H9B	109.5	C19—C18—C17	120.3 (2)
H9A—C9—H9B	109.5	C19—C18—H18	119.9
C8—C9—H9C	109.5	C17—C18—H18	119.9
H9A—C9—H9C	109.5	C18—C19—C20	119.2 (2)
H9B—C9—H9C	109.5	C18—C19—H19	120.4
N1—C10—C11	109.45 (15)	C20—C19—H19	120.4
N1—C10—C16	113.53 (16)	C21—C20—C19	120.0 (2)
C11—C10—C16	111.07 (15)	C21—C20—H20	120.0
N1—C10—H10	107.5	C19—C20—H20	120.0
C11—C10—H10	107.5	C20—C21—C16	122.1 (2)
C16—C10—H10	107.5	C20—C21—H21	119.0
C10—C11—C15	113.65 (17)	C16—C21—H21	119.0
C10—C11—C12	115.59 (17)	C10—N1—C7	116.59 (15)
C15—C11—C12	102.53 (18)	C10—N1—H1	108.9 (16)
C10—C11—H11	108.2	C7—N1—H1	113.1 (16)
C15—C11—H11	108.2	C4—O1—H1A	109.5
			
C6—C1—C2—C3	2.6 (3)	C11—C12—C13—C14	−27.7 (3)
Cl2—C1—C2—C3	−176.33 (18)	C12—C13—C14—C15	6.6 (3)
C1—C2—C3—C4	−0.2 (3)	C13—C14—C15—C11	17.1 (3)
C2—C3—C4—O1	177.9 (2)	C10—C11—C15—C14	−159.2 (2)
C2—C3—C4—C5	−2.2 (3)	C12—C11—C15—C14	−33.7 (2)
O1—C4—C5—C6	−177.83 (19)	N1—C10—C16—C17	−122.7 (2)
C3—C4—C5—C6	2.2 (3)	C11—C10—C16—C17	113.4 (2)
O1—C4—C5—C7	−0.6 (3)	N1—C10—C16—C21	58.1 (2)
C3—C4—C5—C7	179.43 (19)	C11—C10—C16—C21	−65.8 (2)
C2—C1—C6—C5	−2.6 (3)	C21—C16—C17—C18	−0.5 (3)
Cl2—C1—C6—C5	176.38 (15)	C10—C16—C17—C18	−179.69 (18)
C4—C5—C6—C1	0.1 (3)	C21—C16—C17—Cl1	178.20 (14)
C7—C5—C6—C1	−177.13 (18)	C10—C16—C17—Cl1	−1.0 (3)
C6—C5—C7—N1	−145.10 (17)	C16—C17—C18—C19	0.0 (3)
C4—C5—C7—N1	37.7 (2)	Cl1—C17—C18—C19	−178.73 (18)
C6—C5—C7—C8	95.3 (2)	C17—C18—C19—C20	0.7 (4)
C4—C5—C7—C8	−81.9 (2)	C18—C19—C20—C21	−0.9 (4)
N1—C7—C8—C9	178.4 (2)	C19—C20—C21—C16	0.4 (3)
C5—C7—C8—C9	−60.7 (3)	C17—C16—C21—C20	0.3 (3)
N1—C10—C11—C15	175.01 (17)	C10—C16—C21—C20	179.5 (2)
C16—C10—C11—C15	−58.8 (2)	C11—C10—N1—C7	179.95 (17)
N1—C10—C11—C12	56.8 (2)	C16—C10—N1—C7	55.2 (2)
C16—C10—C11—C12	−177.03 (17)	C5—C7—N1—C10	71.5 (2)
C10—C11—C12—C13	161.7 (2)	C8—C7—N1—C10	−165.80 (17)
C15—C11—C12—C13	37.5 (2)		

### Hydrogen-bond geometry (Å, °)

**Table:**

*D*—H···*A*	*D*—H	H···*A*	*D*···*A*	*D*—H···*A*
N1—H1···Cl2^i^	0.85 (2)	2.91 (1)	3.7023 (18)	156 (2)
O1—H1A···N1	0.82	1.93	2.642 (2)	144

Symmetry codes: (i) −*x*+1/2, −*y*+2, *z*+1/2.
